# Low Temperature Hall Effect Investigation of Conducting Polymer-Carbon Nanotubes Composite Network

**DOI:** 10.3390/ijms131114917

**Published:** 2012-11-14

**Authors:** Afarin Bahrami, Zainal Abidin Talib, Wan Mahmood Mat Yunus, Kasra Behzad, Mahnaz M. Abdi, Fasih Ud Din

**Affiliations:** 1 Department of Physics, Faculty of Science, University Putra Malaysia, Serdang 43400, Malaysia; E-Mails: afarin.bah@gmail.com (A.B.); mahmood@science.upm.edu.my (W.M.M.Y.); kasra.behzad@gmail.com (K.B.); uddin.fasih@gmail.com (F.U.D.); 2 Department of Physics, Faculty of Science, Islamic Azad University, Eslamshahr Branch, 3314767653, Iran; 3 Department of Physics, Faculty of Science, Islamic Azad University, Shahre Ghods Branch, 3754198811, Iran; 4 Department of Chemistry, Faculty of Science, University Putra Malaysia, Serdang 43400, Malaysia; E-Mail: mahnaz@putra.upm.edu.my

**Keywords:** polypyrrole, carbon nanotube, Hall effect, conductivity

## Abstract

Polypyrrole (PPy) and polypyrrole-carboxylic functionalized multi wall carbon nanotube composites (PPy/*f*-MWCNT) were synthesized by *in situ* chemical oxidative polymerization of pyrrole on the carbon nanotubes (CNTs). The structure of the resulting complex nanotubes was characterized by transmission electron microscopy (TEM) and X-ray diffraction (XRD). The effects of *f*-MWCNT concentration on the electrical properties of the resulting composites were studied at temperatures between 100 K and 300 K. The Hall mobility and Hall coefficient of PPy and PPy/*f*-MWCNT composite samples with different concentrations of *f-*MWCNT were measured using the van der Pauw technique. The mobility decreased slightly with increasing temperature, while the conductivity was dominated by the gradually increasing carrier density.

## 1. Introduction

Carbon nanotubes (CNTs) possess extraordinary properties, such as high Young’s modulus, good flexibility, and high thermal and electrical conductivities [1,2]. Additionally, conducting polymers (CPs) can be highly conjugated and show metal-like conductivity, as well as reversible chemical and physical properties through doping/dedoping processes [3,4]. They are promising nanomaterials for new applications in chemistry and physics, particularly for the development of new devices such as hydrogen storage, supercapacitors, biosensors, electromechanical actuators, nanoprobes for high-resolution imaging, and so on [5,6]. Therefore, the preparation and investigation of the fundamental properties of CNT/CP composites is of interest. In the present study, polypyrrole-carboxylic functionalized multi-wall carbon nanotubes (PPy/*f*-MWCNT) composites were synthesized by *in situ* chemical polymerization of monomers in the presence of varying *f*-MWCNT content.

There are various methods for manufacturing composites based on PPy and CNTs [7–10]. The most common are chemical and electrochemical polymerization of pyrrole in the presence of CNTs [11,12]. MWCNTs can be incorporated into PPy matrices through the first technique by functionalization of nanotubes, and consists of the oxidation of the nanotube side walls by the bonding of carboxylic acid groups, dispersion the nanotubes in pyrrole monomer mixtures, and the subsequent electropolymerization [13] or enzyme-initiated polymerization [14]. PPy/CNT composites were synthesized by Long *et al*. [15] by applying chemical *in situ* oxidative polymerization methods. Moreover, Zhang *et al*. reported size-controlled nanocables using chemical *in situ* oxidative polymerization in the presence of a cetyl trimethyl ammonium bromide cationic surfactant or polyethylene glycol mono-p-nonphenyl ether, which functioned as a non-ionic surfactant [16]. By changing the pyrrole/*f-*MWCNT mass ratio, it was possible to control the thickness of pyrrole layer in PPy/*f*-MWCNT composite [17]. A novel route was introduced by Karim *et al*. for synthesizing PPy/*f*-MWCNT composites using gamma-radiation-induced *in situ* chemical polymerization [18]. Furthermore, for synthesizing composite materials with *f*-MWCNTs, the interfacial polymerization of pyrrole has also been employed [19].

Hall effect measurements are important for semiconductor material characterization due to the information that can be obtained, which includes Hall voltage, the conductivity type, carrier density, and mobility. In addition, measuring mobility is especially important for designing efficient transistor switching and efficient charge separation in photovoltaic devices. Mobility strongly depends on the nature of the material, its structure, and purity. Generally, the mobility of holes is significantly lower than that of electrons in inorganic semiconductors; however, for organic semiconductors the mobility of positive polarons is significantly higher.

We investigated the low temperature Hall effect and conductivity of PPy and PPy/*f*-MWCNT composites prepared with *in situ* chemical polymerization. The 3-dimensional (3-D) variable range hopping model proposed by Mott [20,21] provides the best fit to the conductivity *versus* temperature data, and with increasing the weight percentage of *f*-MWCNTs the value of the Mott’s hopping parameter (*T*_0_) decreased by two orders of magnitude. Furthermore, the Hall mobility decreases slightly with temperature, while the conductivity is dominated by the gradually increasing carrier density.

## 2. Results and Discussion

**Figure 1:**
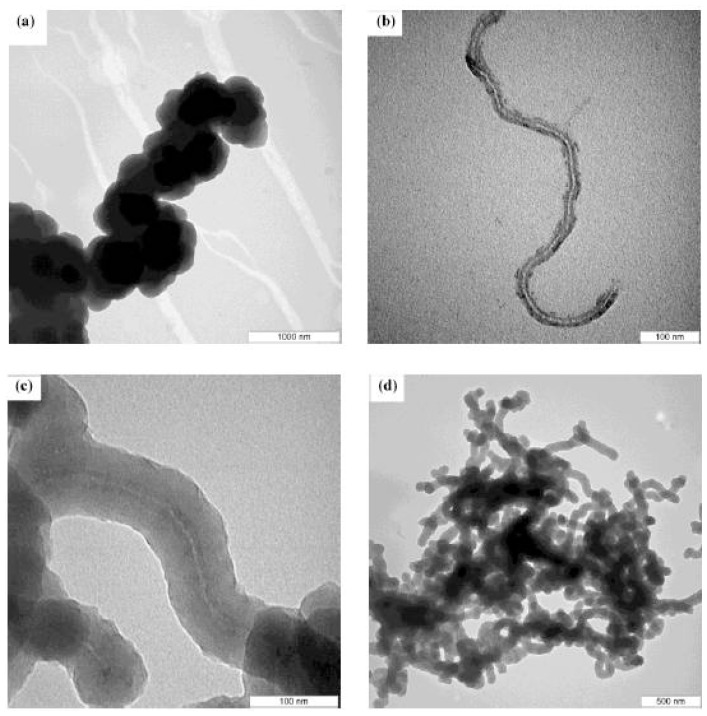
TEM photographs of (**a**) Polypyrrole (PPy); (**b**) functionalized multi wall carbon nanotube composites (*f*-MWCNT); (**c**) and (**d**) PPy coated *f*-MWCNTs at different magnifications.

Figure 1a to 1d show TEM images of the PPy formed by chemical polymerization without *f*-MWCNT, functionalized MWCNTs and the PPy/*f*-MWCNT composite formed by *in situ* chemical polymerization, respectively. The TEM images clearly show that the surface of CNTs is coated with PPy formed on the surface of *f*-MWCNT by oxidative polymerization.

**Figure 2:**
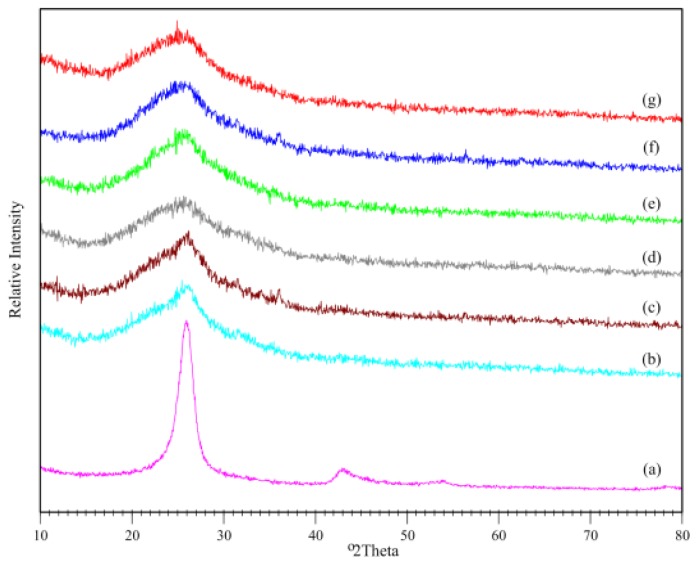
X-ray diffractograms of (**a**) *f*-MWCNT and PPy/ *f*-MWCNT composites with (**b**) 20%; (**c**) 16%; (**d**) 12%; (**e**) 8%; (**f**) (4%) and (**g**) 0% *f*-MWCNTs.

Figure 2 shows XRD spectra for *f*-MWCNTs, PPy and PPy/*f*-MWCNT nanotubes at different weight ratios of *f*-MWCNTs. Two diffraction peaks exist at 26° and 44° for the *f*-MWCNTs, and correspond to a graphite-like [22] structure. No crystalline peaks appeared for PPy, indicating its amorphous structure. The XRD data of the composites revealed spectrum similar to those for pure PPy, indicating that no additional crystalline order or chain arrangement was introduced into the composites. This result shows that a PPy layer coated the surface of the *f*-MWCNTs. As expected, the intensity of the XRD peaks decreased with a decrease in the weight percentage of *f*-MWCNTs to pyrrole.

**Figure 3:**
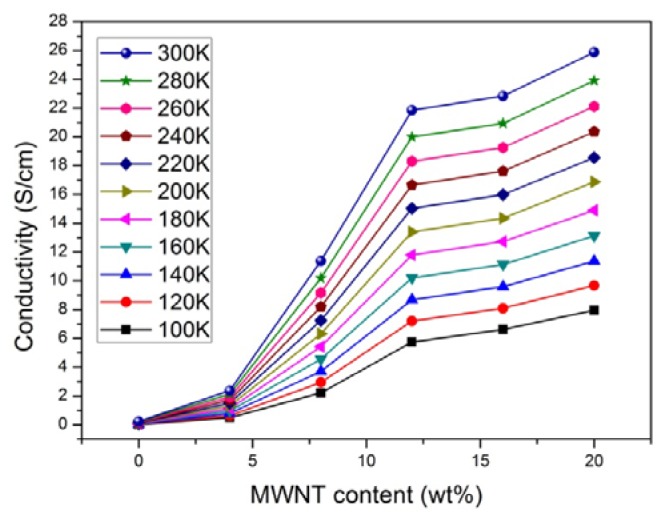
The conductivity of PPy and PPy/*f*-MWCNT composites, at different temperatures, as a function of *f*-MWCNT content.

Figure 3 shows the conductivity (σ) of PPy and PPy/*f*-MWCNT composites of CNTs as a function of *f*-MWCNT content with increased temperature. The increasing conductivity of the composite with CNT weight percentage and temperature is clearly observed.

The room temperature (300 K) conductivity σ of the PPy was 0.235 S/cm, while for 20% composite it was 25.879 S/cm. For comparison, the σ (300 K) value of the pressed pellet of CNTs was about 1.95 S/cm. The more than two orders of magnitude increase in σ indicates the percolative behavior.

**Figure 4:**
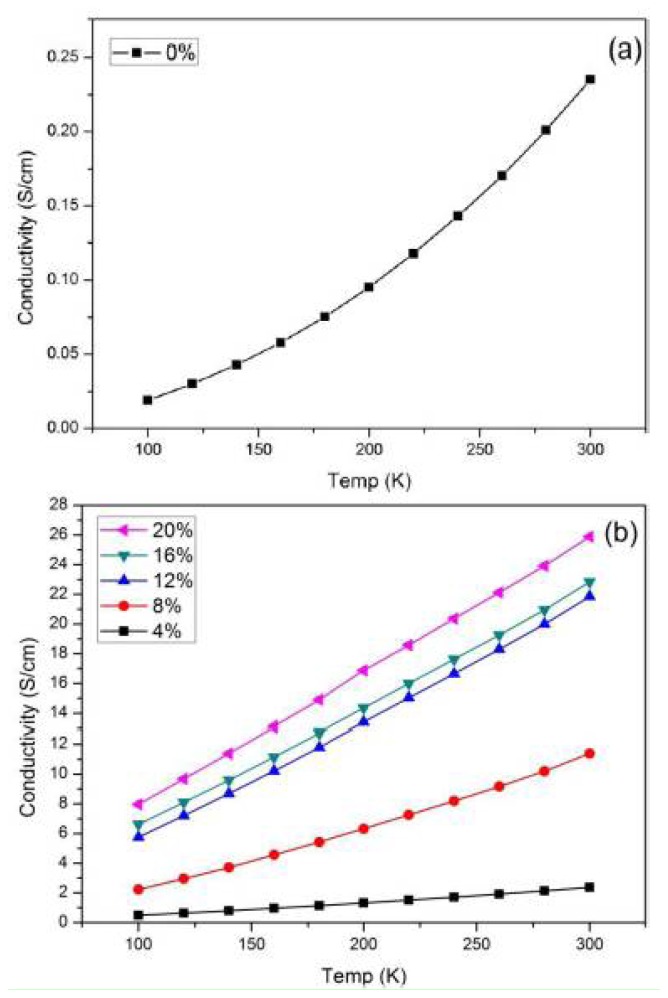
The conductivity of (**a**) PPy and (**b**) PPy/*f*-MWCNT composites at temperatures between 100 K and 300 K.

The increase in σ (300 K) as a function of the CNT mass fraction is directly related to the introduction of conducting CNT pathways to the polymer, and is indicative of percolative behavior. It may also be due to charge transfer from PPy to *f*-MWCNTs and the improved compactness of PPy by *f*-MWCNTs [23,24]. The intrinsic carrier concentration in CPs is also related to π-conjugation length and doping degree, thus, increasing the *f*-MWCNT content will lead to an increase in both the conjugation length of the polymer and the carrier concentration with *f*-MWCNT content. Therefore, to obtain more insight on the conduction mechanism and to verify how the CNTs affect the composite conductivity, the dependence of conductivity on temperature was examined, as shown in Figure 4.

The conductivity of PPy and PPy/*f*-MWCNT increases with an increase in temperature, showing a characteristic semiconductor behavior. To explain the electrical response of the composite, variable range hopping (VRH) model, proposed by Mott [20], was employed as given by the equation below:

$$\sigma_{\text{dc}}=\sigma_{0}\;\text{exp }[-{\left(T_{0}/T\right)}^{1/d+1}]$$

where σ_0_ is a constant, *T*_0_ = 24/[π *k*_B_*L*_C_^3^*N* (*E*_F_)] is the characteristic Mott temperature that depends on the hopping parameter, *T* is the Kelvin temperature, *k*_B_ is the Boltzmann constant, *L*_C_ is the localization length, *N* (*E*_F_) is the density of the states at the Fermi level, and *d* is the dimensionality of the conduction process.

**Figure 5:**
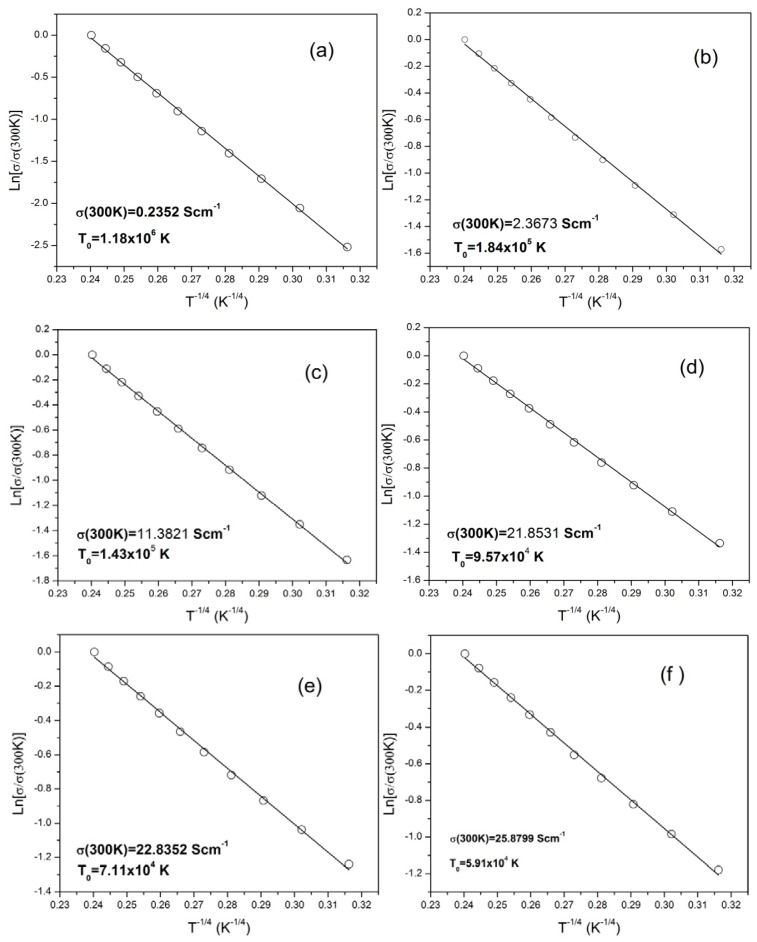
Fitting of the electrical conductivities of the PPy/*f*-MWCNT composites with Mott’s VRH model equation: (**a**) 0%; (**b**) 4%; (**c**) 8%; (**d**) 12%; (**e**) 16%; and (**f**) 20%.

The 3-dimensional (3D) VRH model provided the best fit to the data, as shown in the Figure 5, and the Mott’s equation becomes:

$$\sigma_{\text{dc}}=\sigma_{0}\;\text{exp }[-{\left(T_{0}/T\right)}^{1/3+1}]$$

Figure 5 shows the electrical conductivity plotted for Ln (σ*/*σ (300K)) *versus* (*T*^−1/4^). The pattern of the plots shows a significant temperature and dopant concentration dependence. The solid lines represent the best fit of Mott’s equation to the experimental data.

The values of the linear regression *R*^2^ calculated for the different mechanisms are given in Table 1, and show that the most suitable mechanism is the 3D VRH transport.

The *T*_0_ values for the six samples are calculated from the slope of the lnσ (*T*) *versus T*^−1/4^ plot and shown in Table 2. From Table 2, it is clear that with increasing the weight percentage of *f*-MWCNTs the value of the hopping parameter (*T*_0_) decreased by two orders of magnitude, which is an indication that the barrier height of the composite is reduced when the *f*-MWCNT content is increased. In other words, the charge delocalization in PPy/*f*-MWCNT composite increases with *f*-MWCNT content.

**Figure 6:**
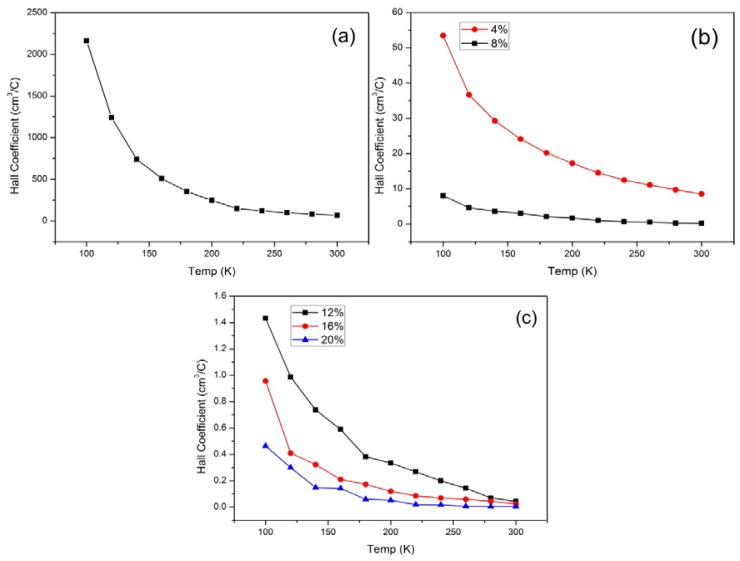
Temperature dependence of the Hall coefficient for (**a**) PPy; (**b**) PPy/*f*-MWCNT with 4 and 8 weight percentage of *f*-MWCNT and (**c**) PPy/*f*-MWCNT with 12, 16 and 20 weight percentage of *f*-MWCNT.

In addition to the conductivity, the Hall coefficient of the samples as function of temperature was also measured. A Hall field (fixed at 0.5 *T* for the entire temperature range) orthogonal to the current density was applied. The results are shown in Figure 6. The Hall coefficient for 20 wt% *f*-MWCNT concentration increased by a factor of 2, upon cooling from 300 K to 100 K.

The Drude model was used to determine the carrier concentration from the Hall coefficient. Assuming one carrier conduction theory, the carrier density, *P*_c_, and Hall mobility μ_H_ can be calculated from:

$$P_{\text{c}}={\left(e\;R_{\text{H}}\right)}^{-1}\;\text{and }\mu_{\text{H}}=R_{\text{H}}\;\sigma$$

where *e* is the electronic charge and R_H_ is the Hall coefficient. The interpretations given use a two-band conduction model [25] in which the maximum in *R*_H_ (*T*) is explained by the crossover from conduction band to impurity band when T decreases. For 20 wt% *f*-MWCNT concentration at 300 K, yields a carrier concentration of 1.99 × 10^21^ cm^−3^, a value that is also reduced at the same rate as the increase in R_H_ toward low temperature. It can be determined that the carrier density increases by approximately 2 orders of magnitude between 100 K and 300 K. This behavior suggests that the system is a semiconductor. The reduction of carrier density with decreasing temperature was common among all the samples.

Mobility is an important parameter, both for understanding device performance as well as studying the underlying semiconductor physics in these materials. The charge carrier transport in organic compounds occurs within molecules, between molecules, as well as between crystal planes and grains. These multiple ‘barriers’ strongly inhibit charge carrier transport; hence, the mobility of organic semiconductors is significantly lower than that of inorganic ones. The carrier mobility in organic molecular crystals is generally quite low, because it is difficult for carriers to move from molecule to molecule due to poor intermolecular overlap.

**Figure 7:**
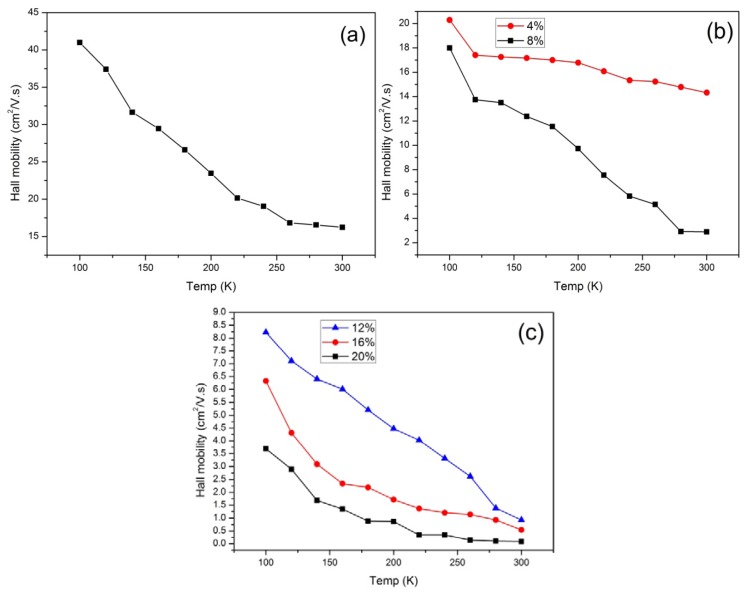
Temperature dependence of the Hall mobility for (**a**) PPy; (**b**) PPy/*f*-MWCNT with 4 and 8 weight percentage of *f*-MWCNT and (**c**) PPy/*f*-MWCNT with 12, 16 and 20 weight percentage of *f*-MWCNT.

From Figure 7, for all the samples the Hall mobility is roughly inversely proportional to T. The Hall mobility decreased with increasing temperature, however, it did not inhibit the increase in the carrier concentration, and the conductivity increased with increasing temperature. In other words, although the carrier mobility decreased with increasing temperature, it was compensated by the increasing carrier concentration with temperature. These two factors compete during the rise in temperature. Because the conductivity increases with temperature, variation in carrier concentration is more important than changes in the mobility.

**Figure 8:**
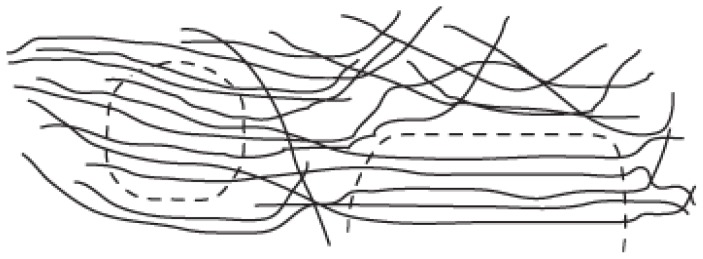
Schematic of the ordered and disordered regions [26].

The carrier density estimated from the Hall coefficient *P*_c_*=* (*e R*_H_)^−1^ for most samples enhanced by approximately 2 orders of magnitude from 100 K to 300 K, while the carrier mobility calculated from the Hall coefficient (μ_H_ = *R*_H_ σ) and the conductivity σ decreases less than 100%. This can explain the decreasing Hall mobility with temperature causing the the conductivity of the samples to still increase. From Figure 7, the Hall mobility is roughly inversely proportional to *T*. The interpretation of the results is complicated because, as shown in Figure 8, generally the polymer contains a mixture of metallic (ordered) and insulating (disordered) regions. How the conductivity is affected by the molecular mobility is also important. The conductivity increased with temperature for PPy and PPy/*f*-MWCNT. The temperature dependence of the Hall mobility can be explained by the reduction of the polymer chains coplanarity (or decrease in degree of crystallinity) that causes the decrease in Hall mobility. The increasing conductivity with temperature is a property of semiconductors, whereas decreasing conductivity is atypical of metals. This temperature dependence supports the two-phase model of CPs with metallic islands embedded in an insulating matrix.

The degrees of disorder, trapping and detrapping also play a significant role in determining the mobility; moreover, the experimental values of the mobility are highly sensitive to the method of preparation of the polymer. Even the inverse relationship shows that it is unclear whether the mobility is only due to hopping. In some cases the chains were long and the mobility might have a drift component [27]. In this temperature range, the Hall mobility decreased with temperature. The inverse relationship of temperature dependence of Hall mobility could suggest the scattering mechanisms are the limiting factor. It may be that the higher temperature caused an increase in lattice (and backbone) vibration that produces more collisions between phonons and carriers and as a result mobility decreased. Furthermore it is important to notice that the decrease in the Hall mobility with increasing temperature is due to the increase of the free carrier generation by temperature that would cause more collision between charge carriers and decreasing the Hall mobility.

The charge carrier mobility in the CPs is usually affected by morphology, crystallinity and charge transport. The changes in the mobility and carrier density are uncorrelated. The results suggest the mechanism that determines the temperature dependence of the mobility and the conductivity is not same. The low values of the Hall effect mobility of polymer materials are usually because of the high degree of disorder and the large density of chemical impurities, as the conductivity is affected by the molecular mobility.

## 3. Experimental Section

The pyrrole monomer (Fluka) was distilled prior to use and stored at 4 °C. *f*-MWCNT (Nanostructured & Amorphous Materials, Inc.) and ferric chloride 6-hydrate (HmbG chemical) were analytical grade and used without further purification. The outside and inside diameters of f-MWCNT were 8–15 nm and 3–5 nm respectively. The length and purity of nanotubes were 10–50 μm and >95%. PPy coated *f*-MWCNT was synthesized by *in-situ* polymerization of pyrrole on *f*-MWCNT. The *f*-MWCNTs with different weight ratios were dispersed in distilled water and sonicated for 4 h to obtain a well dispersed suspension to enhance the disaggregation of any nanotubes bundles. Thereafter, a calculated amount of pyrrole was added to this solution and stirred for 0.5 h. Ferric chloride 6 hydrates (FeCl_3_ 6H_2_O) was added drop wise to the above solution with constant stirring at ambient temperature, and the mixture was stirred again for 1h (the Fe^3+^/pyrrole molar ratio was 2.3). After the reaction, the precipitated PPy/*f-*MWCNTs powders were filtered. The PPy/*f*-MWCNT composite samples were then washed with distilled water and methanol several times until a colorless filtrate was obtained. The resultant product in powder form was vacuum dried at 40 °C for 24 h. The samples were then ground into fine powder and pressed into very thin pellets. The weight percentages of *f*-MWCNTs in the PPy/*f*-MWCNT composites were 0, 4, 8, 12, 16 and 20 wt%.

The structures of the composites were characterized using transmission electron microscopy (TEM) with a HITACHI H-7100. X-ray diffraction (XRD) of the composites was studied using PANalytical X’pert PRO X-ray diffractometer with Cu *K*_α_ targets at a scan rate of 4°/min. Lake Shore Model 7504 Hall measurement system was used for temperature dependent measurements in the temperature range of 100 K to 300 K and in 5 KG magnetic field strengths. The conductivity and Hall effect parameters were measured using standard van der Pauw geometry on circular samples with radius of 13 mm. Silver wires with diameter of 15 μm were attached to the sample using silver paste. The measurements were performed by applying a constant current (10 mA) across a pair of silver wires and the voltage drop was measured across the other electrode pair.

## 4. Conclusions

We have synthesized PPy and PPy/*f*-MWCNT composites using chemical *in-situ* polymerization. Based on TEM and XRD analysis, amorphous PPy completely coats *f*-MWCNTs. The conductivity, carrier concentration and mobility of pure PPy and PPy/*f*-MWCNT composite were measured using the Hall effect technique, from 100 K to 300 K. Similar to other semiconductor materials (organic and inorganic), the carrier density increased with temperature. However, the Hall mobility decreased with temperature, yet increasing the carrier density in this range of temperature had a compensating effect, to yield an overall increase in the conductivity. The Hall voltage measurements and consequent parameters derived demonstrate the composite’s potential applications in both harvesting solar energy and optoelectronic applications. *f*-MWCNTs play a key role in the composites during carrier transfer in the temperature range of 100K to room temperature. The Hall coefficient graphs indicate that the *f*-MWCNT content increased the carrier density, due to the increased conjugation length of CNTs. These characteristics of enhanced electrical properties provide new opportunities for potential applications of these composites in a number of scientific and technological fields.

## Figures and Tables

**Table 1:** Calculated values of linear regression with different mechanisms for PPy and PPy/*f***-**MWCNT with different percentages of nanotubes.

Transport mechanism	Linear regression, *R*^2^					

	*f*-MWCNT content					

	0%	4%	8%	12%	16%	20%
1D	0.99556	0.99297	0.99532	0.99399	0.99201	0.99322
2D	0.9984	0.99681	0.99829	0.99748	0.99615	0.99696
3D	0.99927	0.99816	0.99922	0.99867	0.99766	0.99827

**Table 2:** Experimental values of the room temperature conductivity, σ (300 K), and *T*_0_ for PPy and PPy/*f*-MWCNT composite.

Sample	0 wt%	4 wt%	8 wt%	12 wt%	16 wt%	20 wt%
σ (S/cm)	0.235	2.37	11.4	21.9	22.8	25.9
*T*^0^ (K)	1.18 × 10^6^	1.84 × 10^5^	1.43 × 10^5^	9.57 × 10^4^	7.11 × 10^4^	5.91 × 10^4^
